# Supplementary material for: FA Sliding as the Mechanism for the ANT1-Mediated Fatty Acid Anion Transport in Lipid Bilayers
Source: Int J Mol Sci. 2023 Sep 5;24(18):13701. doi: 10.3390/ijms241813701 (PMC10531397; doi:10.3390/ijms241813701)
Supplement: Supplementary file 1 [file ijms-24-13701-s001.zip › 230725_Table 1.pdf]

Supplementary Table S1: Exchange rates of ANT1 and ANT1 mutants calculated from the time-course of ^{3}H-ATP release measured in liposomes as displayed in Supplementary Fig. 1.

**Protein**	**Exchange rate, μmol ATP per mg protein per minute**	**Exchange rate, Relative to ANT1**
ANT1	114 ± 5	1.000 ± 0.062
ANT1K22S	70.1 ± 2.8	0.615 ± 0.036
ANT1K48S	83.8 ± 17.1	0.735 ± 0.153
ANT1K51S	94.9 ± 13.7	0.832 ± 0.126
ANT1R59S	53.5 ± 6.8	0.469 ± 0.063
ANT1K62S	120 ± 2	1.052 ± 0.049
ANT1R79S	25.0 ± 3.1	0.219 ± 0.029
ANT1K93S	97.1 ± 8.1	0.852 ± 0.080
ANT1D134S	104 ± 19	0.912 ± 0.171
ANT1R137S	103 ± 49	0.904 ± 0.432
ANT1R279S	119 ± 28	1.044 ± 0.250
