# Supplementary material for: FA Sliding as the Mechanism for the ANT1-Mediated Fatty Acid Anion Transport in Lipid Bilayers
Source: Int J Mol Sci. 2023 Sep 5;24(18):13701. doi: 10.3390/ijms241813701 (PMC10531397; doi:10.3390/ijms241813701)
Supplement: Supplementary file 1 [file ijms-24-13701-s001.zip › 230903_Suppl_Figures_FA_Transport_revision_final.pdf]

Supporting information

FA Sliding as the Mechanism for the ANT-mediated Fatty Acid Anion Transport in Lipid Bilayers

- Jürgen Kreiter[1,§,$], Sanja Škulj[1,$], Zlatko Brkljača[2,&], Sarah Bardakji[1], Mario Vazdar[3,*], Elena E. Pohl[1,*]
- [1]Institute of Physiology, Pathophysiology, and Biophysics, Department of Biomedical Sciences, University of Veterinary Medicine, Vienna, Austria
- [2]Division of Organic Chemistry and Biochemistry, Rudjer Bošković Institute, Zagreb, Croatia
- [3]Department of Mathematics, Informatics, and Cybernetics, University of Chemistry and Technology, 166 28 Prague, Czech Republic
- [§]Present address: Institute of Molecular and Cellular Physiology, Stanford Medical School, Stanford, CA, USA
- [&]Present address: Selvita d.o.o., Prilaz baruna Filipovića 29, 10000 Zagreb Croatia
- [$]Equally contributed authors
- *Corresponding authors: elena.pohl@vetmeduni.ac.at (E.E.P.); mario.vazdar@vscht.cz (M.V.)

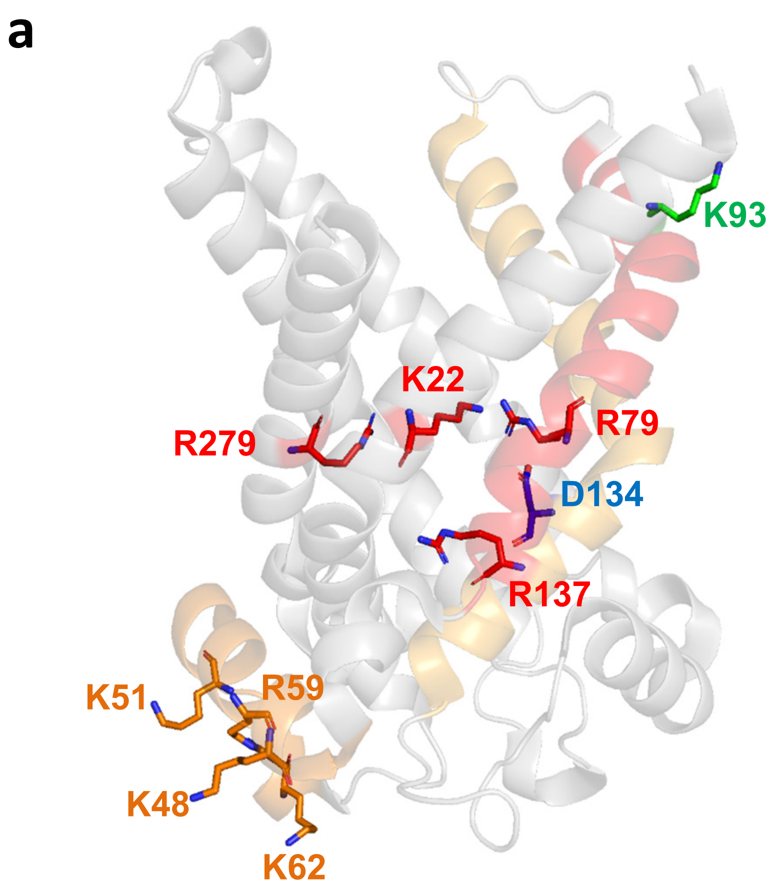

Supplementary Fig. S1. Overview of the location of amino acids mutated in this study.
(a) Structure of ANT1 (PDB:1OKC) viewed from the side. Helix 2 and 3 are colored in red and yellow, respectively. Amino acids K48, K51, R59 and K62 involved in the FA binding (in licorice) are colored in orange, K22, R79, R137 and R279 from the substrate binding site - in red, D134 as a proton donor - in blue, and K93 at the intermembrane space - in green. (b) Side view of the mutated residues in helix 2 (R79 and K93) and helix 3 (D134 and R137).

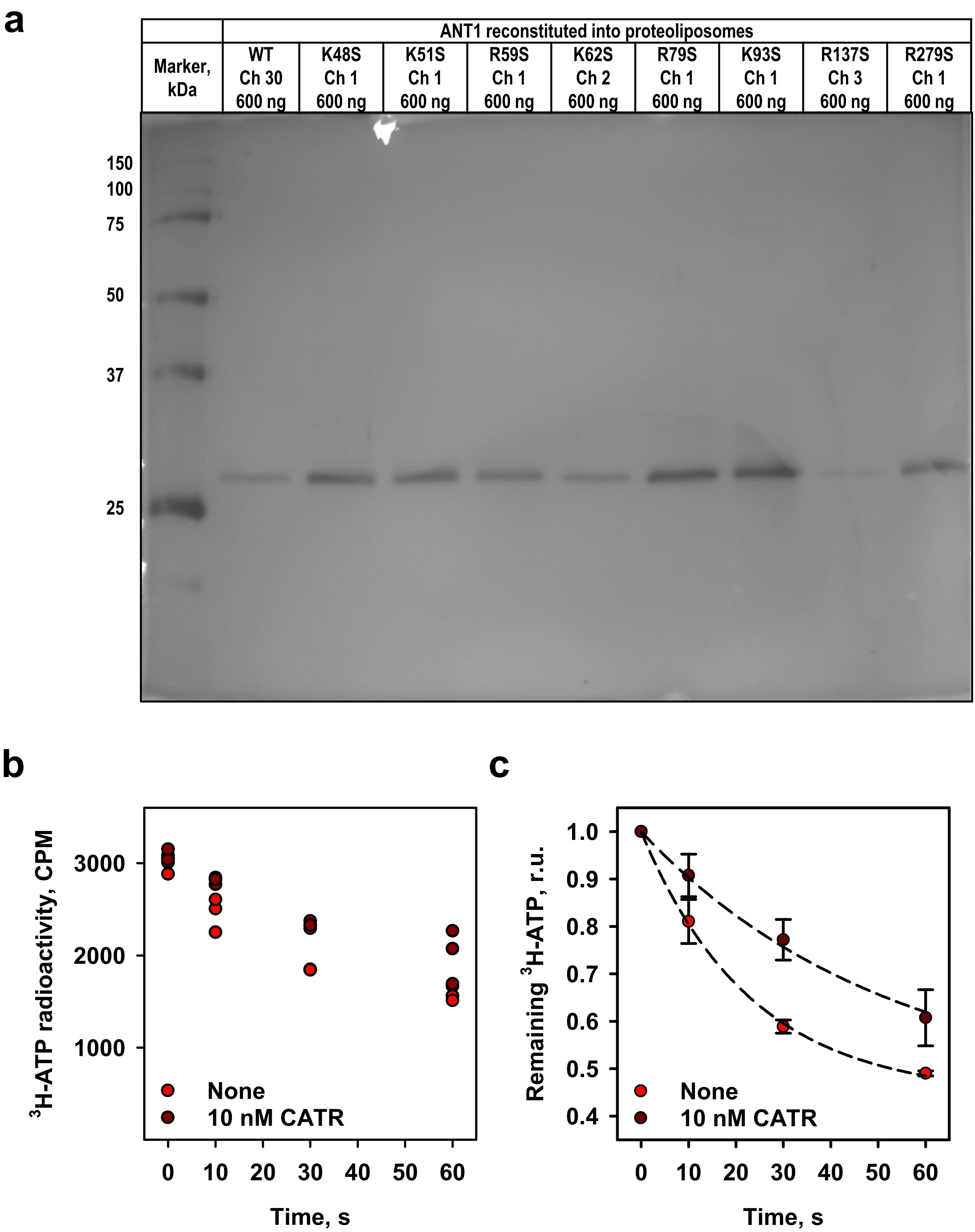

Supplementary Fig. S2.

Supplementary Fig. S2.

(a) Silverstaining of the recombinant ANT1 WT and mutants in proteoliposomes. Protein content was 600 ng.

(b, c) ANT1-mediated ADP/ATP transport and it´s inhibition by carboxyatractyloside (CATR).

Time course of the 3H-ATP concentration in liposomes in absolute (radioactivity counts per minute (CPM)) (b) and relative (c) units in the presence of ANT1 and no CATR (red circles) and in the presence of 10 nM CATR (dark red circles). Lines in (c) represent a least square regression fit of an exponential function to the data. In all experiments, the concentration of ADP and ATP were 2 mM, the lipid concentration was 1.5 mg/ml and the protein concentration was 8.5 - 9 µg/(mg of lipid). Membranes were made of PC:PE:CL (45:45:10 mol%). Buffer contained 50 mM Na_2SO_4, 10 mM Tris, 10 mM MES and 0.6 mM EGTA at pH = 7.34 and T = 296 K. ATP and ADP were dissolved in buffer solution and adjusted to pH = 7.34. Data are shown as the mean ± SD from at least three independent experiments.

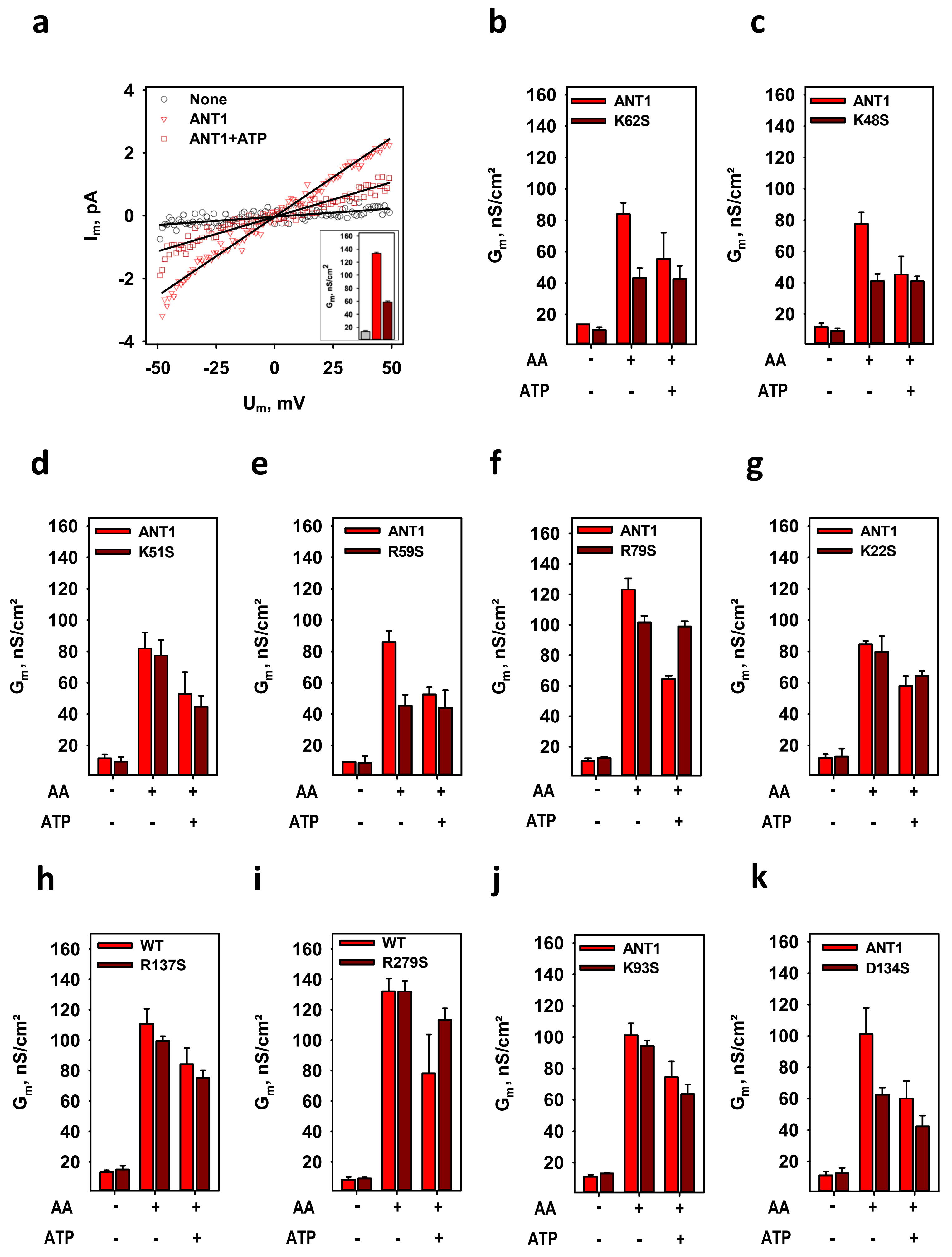

Supplementary Fig. 3

Supplementary Fig. S3. Electrical parameters of planar lipid bilayers reconstituted with ANT1 and mutants.

(a) Representative current-voltage recordings of lipid bilayer membranes reconstituted with ANT1 in the absence of arachidonic acid (AA, black circles), in the presence of AA (red triangles) and in the presence of AA and 2 mM ATP (dark red squares). Insert: The total membrane conductance (G_m) is determined from the slope of a linear fit to the data. (b - k) G_m of the planar lipid bilayers reconstituted with the ANT1-K22S (b), ANT1-K48S (c), ANT1-K51S (d), ANT1-R59S (e), ANT1-K62S (f), ANT1-R79S (g), ANT1-K93S (h), ANT1-D134S (i), ANT1-R137S (j) and ANT1-R279 (k) and parallel refolded ANT1 in the absence of AA or in the presence of AA or in the presence of AA and 2 mM ATP (third data set). In all measurements, lipid concentration was 1.5 mg/ml and the protein concentration - 4 µg/(mg of lipid). Membranes were made of PC:PE:CL (45:45:10 mol%) reconstituted with AA in concentration indicated in the figures. Buffer contained 50 mM Na_2SO_4, 10 mM Tris, 10 mM MES and 0.6 mM EGTA at pH = 7.34 and T = 306 K. ATP was dissolved in buffer solution and adjusted to pH = 7.34. Data are displayed as the mean ± SD of at least three independent measurements.

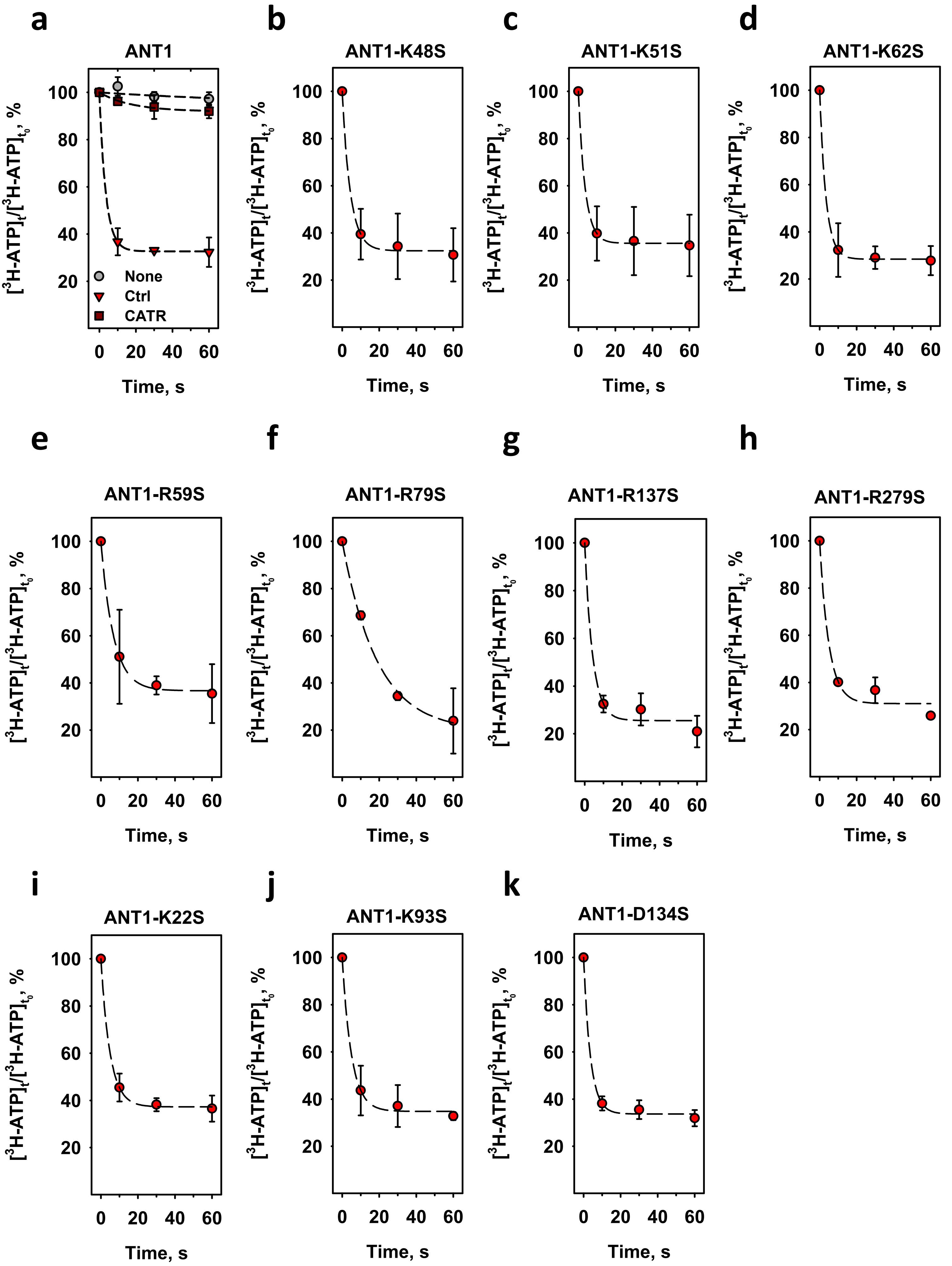

Supplementary Fig. S4

Supplementary Fig. S4. Release of ^{3}H-ATP from liposomes reconstituted with ANT1 or mutants.

Time course of the ^{3}H-ATP concentration in liposomes in the absence (grey circles) and in the presence of reconstituted ANT1 (red) (a), ANT1-K22S (b), ANT1-K48S (c), ANT1-K51S (d), ANT1-R59S (e), ANT1-K62S (f), ANT1-K93S (g), ANT1-R79S (h), ANT1-D134S (i), ANT1-R137S (j) or ANT1-R279S (k). Concentration of CATR in (a) was 100 μM (dark red). Lines represent a least square regression fit of an exponential function to the data. In all experiments, the lipid concentration was 1.5 mg/ml and the protein concentration was 8.5 - 9 μg/(mg of lipid). Membranes were made of PC:PE:CL (45:45:10 mol%). Buffer contained 50 mM Na_2SO_4, 10 mM Tris, 10 mM MES and 0.6 mM EGTA at pH = 7.34 and T = 296 K. ATP and ADP were dissolved in buffer solution and adjusted to pH = 7.34. Data are shown as the mean ± SD from at least three independent experiments.

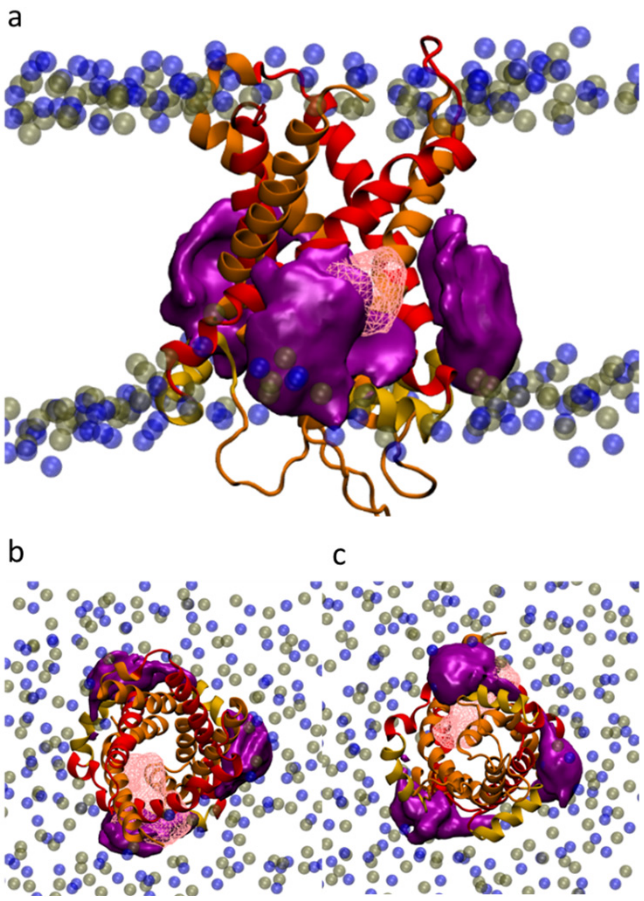

Supplementary Fig. S5. Average volume of cardiolipin and FA^- in 1 μs MD simulation: a) side view, b) top view from cytosol and c) bottom view from the matrix side. Average volumes of cardiolipin and FA^- are presented in violet color and pink wireframe respectively. Odd and even numbered helices are colored in orange and red, respectively. N and P atoms of lipids are colored in blue and green spheres .

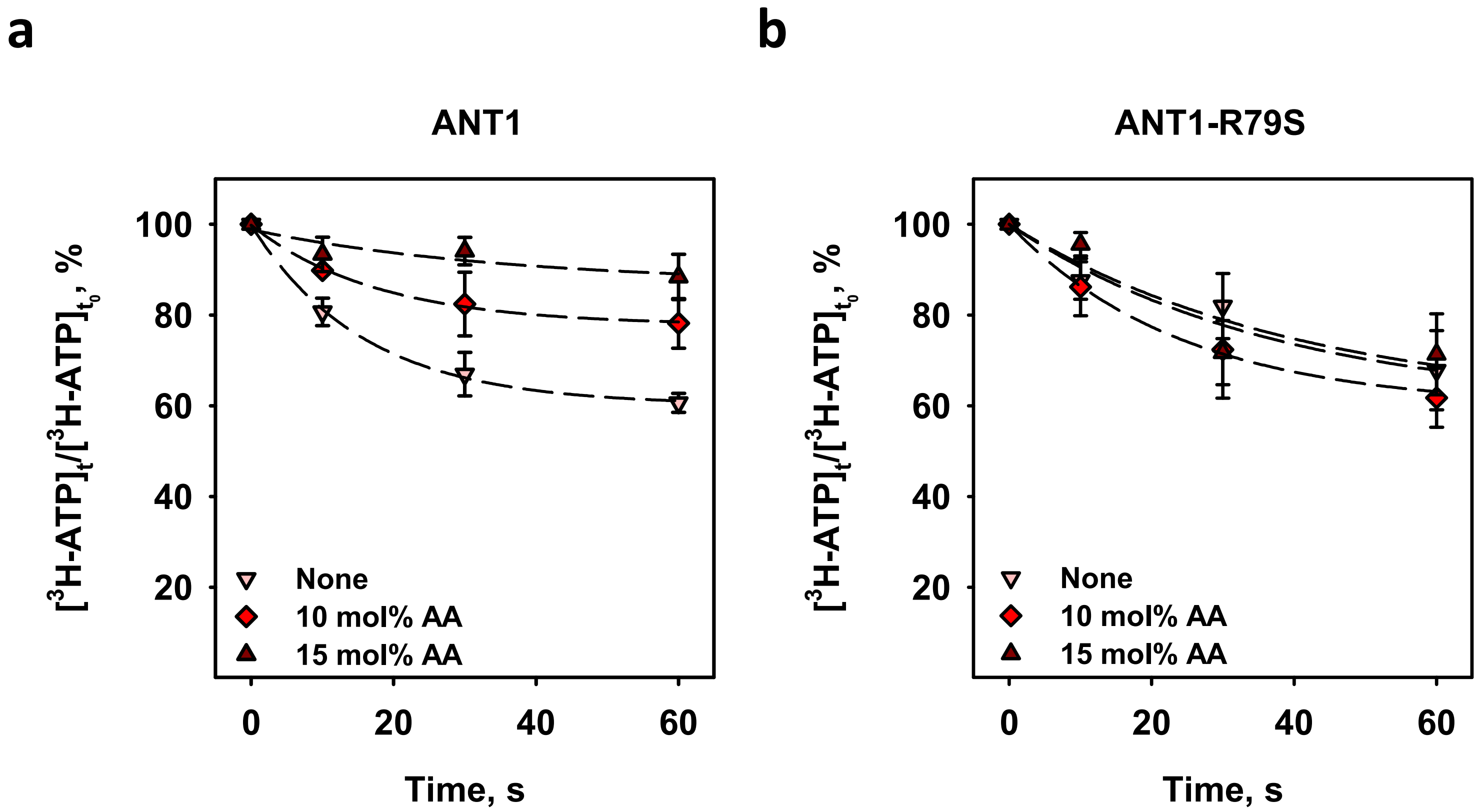

Supplementary Fig. S6. Release of ^{3}H-ATP from liposomes reconstituted with ANT1 or ANT1-R79S.

Time course of the relative ^{3}H-ATP concentration measured in proteoliposomes reconstituted with ANT1 (a) or ANT1-R79S (b) in the absence (none) or presence of 10 or 15 mol% AA. Lines are a least square regression fit of an exponential function to the data.

In all experiments, the lipid concentration was 4 mg/ml and the protein concentration was 4 µg/(mg of lipid). Membranes were made of PC:PE:CL (45:45:10 mol%) reconstituted with AA as indicated in the figures. Buffer solution contained 50 mM Na_2SO_4, 10 mM Tris, 10 mM MES and 0.6 mM EGTA at pH = 7.34 and T = 296 K. Data are the mean ± SD of at least three independent experiments.

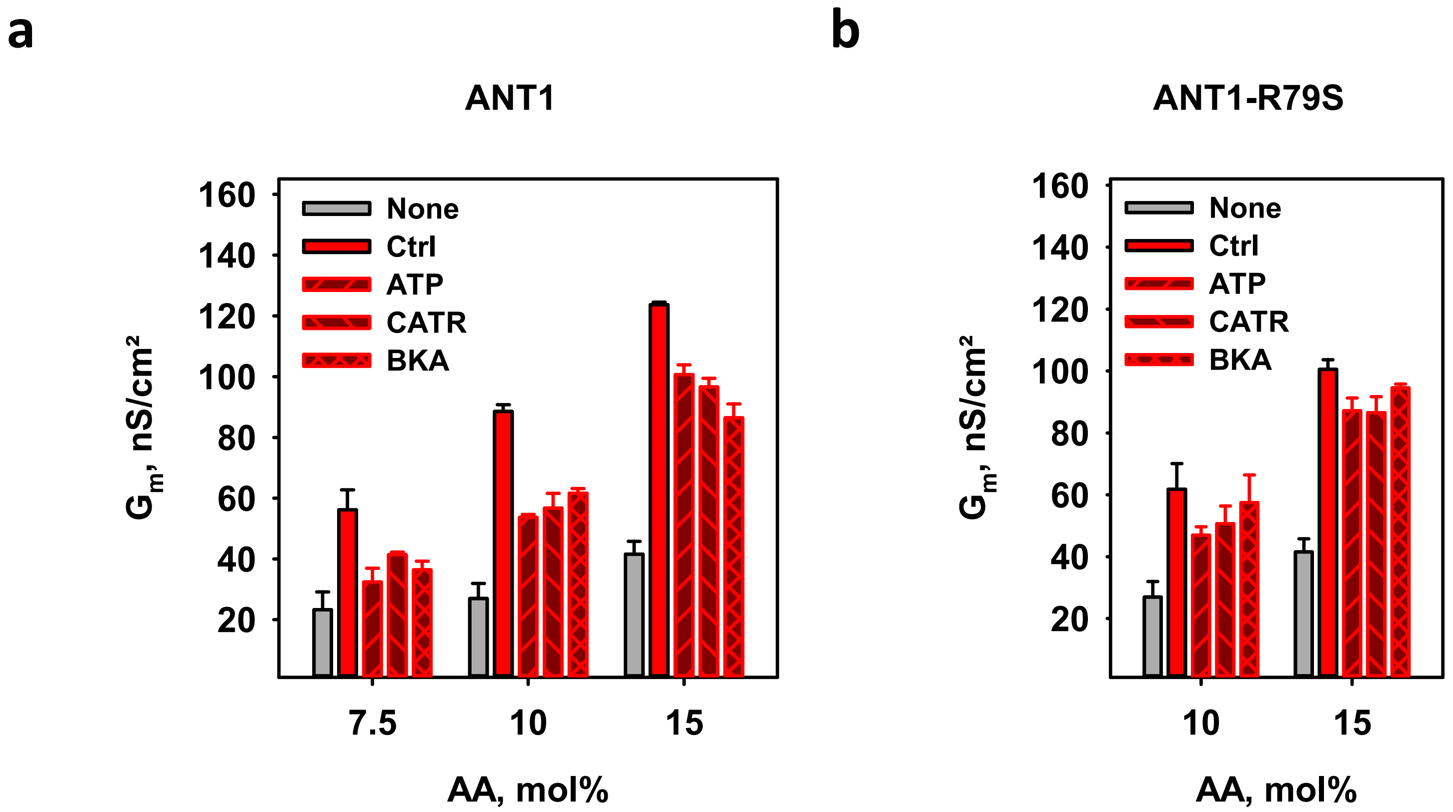

Supplementary Fig. S7. Total membrane conductance of planar lipid bilayers reconstituted with ANT1 or ANT1 R79S

Total membrane conductance (G_m) of planar lipid bilayers reconstituted with ANT1 (a) or ANT1-R79S (b) in the presence of 7.5, 10 or 15 mol% AA. Two control experiments were made in the absence of ANT1 and inhibitors (none) or in the presence of ANT1 only (ctrl). The inhibitors were added in following concentrations: 100 μM ATP, 10 μM CATR or 10 μM BKA. In all experiments, the lipid concentration was 1.5 mg/ml and the protein concentration was 4 μg/(mg of lipid). Membranes were made of PC:PE:CL (45:45:10 mol%) reconstituted with AA as indicated in the figures. Buffer solution contained 50 mM Na_2SO_4, 10 mM Tris, 10 mM MES and 0.6 mM EGTA at pH = 7.34 and T = 306 K. ATP was dissolved in buffer and pH adjusted to 7.34, CATR and BKA were dissolved in DMSO. Data are the mean ± SD of at least three independent experiments.

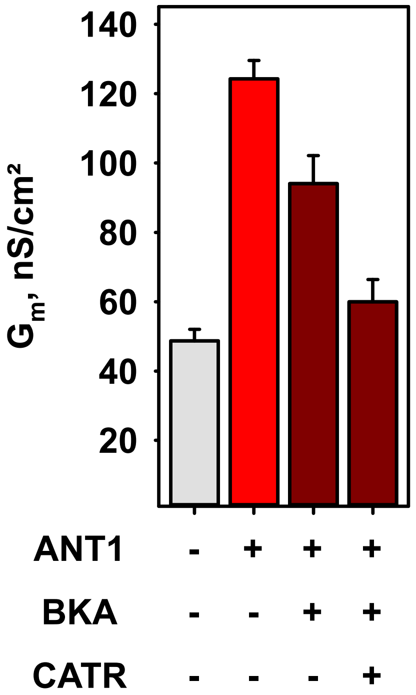

Supplementary Fig. S8. Inhibition of ANT1-mediated FA anion transport by specific inhibitors of ANT1.

Total membrane conductance (G_m) of planar lipid bilayers in the presence of 15 mol% arachidonic acid (AA). BKA and CATR were added in concentration of 10 µM. In all experiments, the lipid concentration was 1.5 mg/ml and the protein concentration was 4 µg/(mg of lipid). Membranes were made of PC:PE:CL (45:45:10 mol%), reconstituted with AA. Buffer solution contained 50 mM Na_2SO_4, 10 mM Tris, 10 mM MES and 0.6 mM EGTA at pH = 7.34 and T = 306 K. BKA and CATR were dissolved in DMSO and subsequently added to the bulk solution. Data are the mean ± SD of at least three independent experiments.
